# Dr. William S. Tremaine

**Published:** 1898-02

**Authors:** 


### Obituary.

Dr. William S. Tremaine, of Buffalo, died at his residence Sunday, January 9, 1898, aged 59 years. He was a native of Canada, but graduated in medicine at the University of Pennsylvania, and entered the military service of the United States during the civil war as assistant-surgeon of the 24th Massachusetts infantry in August, 1863. After serving in various capacities as a medical officer, the details of which will be found in a memorial adopted by the medical society of the county of Erie and published on another page in this issue of the JOURNAL, he was finally retired for disability in February, 1891.

Dr. Tremaine came to Buffalo in 1882, through assignment as post-surgeon at Fort Porter, and has resided here for the most part from that time until his death. During these years he was actively engaged in the practice of surgery and in teaching the science and art thereof for a part of the time at Niagara University. He was ready of speech, interesting and forceful in debate, a skilful operator, resourceful and even original in expedients, and altogether a most useful member of the profession. His widow and five children survive. His funeral was largely attended at St. Paul's cathedral, January 11, 1898, escorted by a battalion of regulars from Fort Porter and his remains were deposited in the vault at Forest Lawn with military honors.

---

MR. ERNEST HART, editor of the *British Medical Journal*, died at his residence in London, January 7, 1898, aged 62 years. His early education was obtained at the City of London school, and his medical education at St. George's hospital medical school, and he was admitted to membership in the Royal College of Surgeons in 1856. Soon afterward he turned his attention to literary work and was in succession assistant editor of the *Lancet*, supervising editor of the *Sanitary Record* and of the *London Medical Recorder*, and finally was appointed editor of the *British Medical Journal* in 1866, holding the latter place until his death. The American profession will recall Mr. Hart's visit to this country in 1893, when he attended the meeting of the American Medical Association at Milwaukee, during which time he addressed the American Medical Editors' Association, and also read a paper in the general session of the American Medical Association on water-borne diseases. Coming again to America in the same year he attended the first Pan-American Medical Congress at Washington, in September, 1893.

A few months ago Mr. Hart underwent amputation of a leg for necrosis complicating diabetes, from which he made a temporary rally, but finally succumbed to the onward progress of the malady. Mr. Hart's fame will largely rest upon the fact that during his editorship of the *British Medical Journal* he lifted it from

